# Therapy-related acute myeloid leukemia following successful treatment of high-risk neuroblastoma in a pediatric patient: a case report and insights into late complications

**DOI:** 10.3389/fonc.2026.1782218

**Published:** 2026-03-03

**Authors:** Si-jia He, Ju Gao, Li-na Qiao, Guo-qian He, Xia Guo, Xiao-yu Jing

**Affiliations:** 1 Department of Pediatrics, West China Second University Hospital, Sichuan University, Chengdu, Sichuan, China; 2 Key Laboratory of Birth Defects and Related Diseases of Women and Children, Ministry of Education, West China Second University Hospital, Sichuan University, Chengdu, Sichuan, China

**Keywords:** allogeneic hematopoietic stem cell transplantation, genetic predispositions, neuroblastoma, secondary neoplasms, therapy-related acute myelomonocytic leukemia

## Abstract

**Background:**

Neuroblastoma is the most common extracranial solid tumor in childhood. With advances in risk-adapted multimodal therapy, survival outcomes for high-risk neuroblastoma have improved substantially. However, prolonged survival has been accompanied by an increasing incidence of therapy-related second malignant neoplasms, which represent a serious late complication and a growing clinical challenge.

**Case presentation:**

We report a rare case of an extremely early-onset secondary malignancy in a young child treated for high-risk neuroblastoma. A 3-year-and-4-month-old girl achieved complete remission after intensive multimodal therapy for stage IV high-risk neuroblastoma. Remarkably, only one month after completion of treatment, she developed therapy-related acute myelomonocytic leukemia (AML, FAB M4 subtype). Bone marrow evaluation revealed high-risk molecular features, including a t(9;11)(p21;q23) translocation resulting in a KMT2A–MLLT3 (MLL/AF9) fusion and concomitant EVI1 overexpression.

**Results:**

The patient was treated with intensive AML-directed chemotherapy followed by allogeneic hematopoietic stem cell transplantation. She achieved complete hematologic and molecular remission, with sustained negativity of minimal residual disease. At the last follow-up in July 2025, she remained in continuous remission for 51 months and had returned to normal school life.

**Conclusion:**

This case highlights an exceptionally short latency of therapy-related AML as a second malignant neoplasm following modern intensive treatment for high-risk neuroblastoma. It underscores the need for heightened vigilance for secondary malignancies even during the early post-treatment period and emphasizes the importance of long-term surveillance strategies in neuroblastoma survivors. Early recognition and timely allogeneic transplantation may offer curative potential in selected high-risk cases. Despite the overall poor prognosis associated with therapy-related acute myeloid leukemia (t-AML), this patient achieved long-term survival following allogeneic hematopoietic stem cell transplantation, highlighting the potential for successful outcomes even in high-risk cases.

## Introduction

Neuroblastoma is the most common extracranial solid tumor in children, comprising 8%–10% of all pediatric malignancies (1). The continuous development of multimodal therapy has driven a remarkable increase in the 5-year survival rate for high-risk disease, from below 20% in the 1970s to more than 50% today (2). The intensive multi-modality treatment strategies currently used for high-risk neuroblastoma, while significantly improving survival, are associated with a markedly increased risk of secondary malignancies, particularly therapy-related acute myeloid leukemia (3, 4). Importantly, t-AML is a heterogeneous entity with distinct biological subtypes. Alkylating agent– and radiation-related t-AML typically develops after a latency of 4–10 years and is often preceded by myelodysplastic syndrome, whereas t-AML associated with topoisomerase II inhibitors is characterized by a shorter latency period, usually 1–5 years, and frequently harbors balanced chromosomal translocations involving KMT2A (MLL) (5).

Current evidence indicates that the risk of therapy-related myelodysplastic and acute leukemia in neuroblastoma patients is primarily influenced by two factors: treatment modalities and genetic predisposition. Regarding treatment, alkylating agents, topoisomerase II inhibitors, and radiotherapy are recognized as critical risk factors (6–9). Studies demonstrate that patients receiving high-dose radiotherapy exhibit a significantly increased incidence of secondary malignancies, often developing within years after initial therapy. Furthermore, balanced chromosomal aberration involving the band 11q23 and disrupting the myeloid/lymphoid leukemia (MLL) gene has been detected in therapy-related myelodysplastic syndrome (t-MDS) and therapy-related acute myeloid leukemia (t-AML) (10). These mutations may alter cellular responses to therapy, impacting tumor recurrence and metastasis.

Consequently, it is imperative to comprehend the genetic predispositions and therapy-associated risk factors contributing to tAML development to facilitate optimal patient surveillance and prompt intervention (11). This report delineates an exceptional case of tAML, characterized by heightened EVI1 expression and the MLL/AF9 translocation, and provides an elucidation of the pertinent literature. While t-AML is generally associated with a poor prognosis due to its therapy-related nature, the successful long-term survival of this patient underscores the possibility of favorable outcomes with timely and appropriate treatment.

## Case presentation

### Clinical manifestations at onset

A 3-year-and-4-month-old girl presented to the Department of Pediatric Hematology and Oncology, West China Second University Hospital with recurrent fever and lymphadenopathy for one month. She had repeated high fevers (peak 39.8 °C) and a firm, poorly mobile left retroauricular lymph node. Subsequently, she developed severe right leg pain preventing ambulation. Initial blood tests at an outside hospital revealed: WBC 8.93×10^9^/L, NEUT% 0.419%, HGB 87 g/L, PLT 333×10^9^/L, CRP 127.14 mg/L. Knee MRI showed bilateral femoral shaft marrow edema with surrounding soft tissue swelling (suggesting osteomyelitis) and a right femoral mass (suggesting enlarged lymph node). Empiric antibiotics (amoxicillin-clavulanate and vancomycin) failed to alleviate her leg pain.

### Diagnosis and treatment of high-risk neuroblastoma

**Figure 1:**
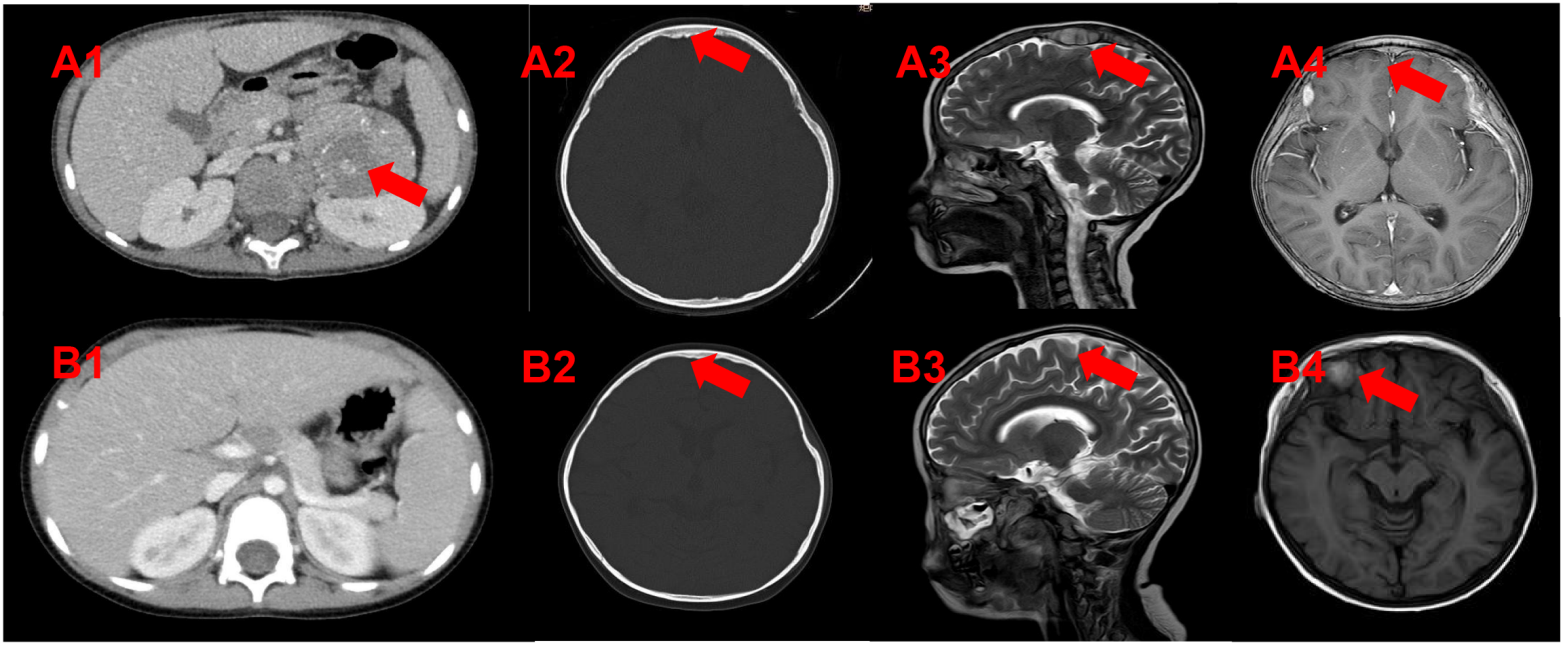
**(A1-A4)** Intra-abdominal and skull lesions at the diagnosis of neuroblastoma. **(B1-B4)** Changes in the intra-abdominal and skull lesions at the diagnosis of therapy-related AML (t-AML).

At our institution, contrast-enhanced CT of the head/thorax/abdomen/knees revealed extensive osteolytic lesions. A heterogeneously enhancing soft tissue mass (about 5.5×4.9×6.9 cm) with calcifications was seen in the left retroperitoneum(**Figure 1A1**). Cranial MRI showed multifocal osseous/cranial lesions, left parietal subdural enhancement with parenchymal involvement, and bilateral frontoparietal dural thickening/nodular enhancement (suspicious for calvarial/meningeal metastases) (**Figures 1A2-A4**). Serum NSE was elevated at 123.7 ng/ml. Bone marrow NB-MRD was <0.01%. N-MYC amplification was negative. The final diagnosis was stage IV, high-risk neuroblastoma on October 1, 2019.

**Figure 2:**
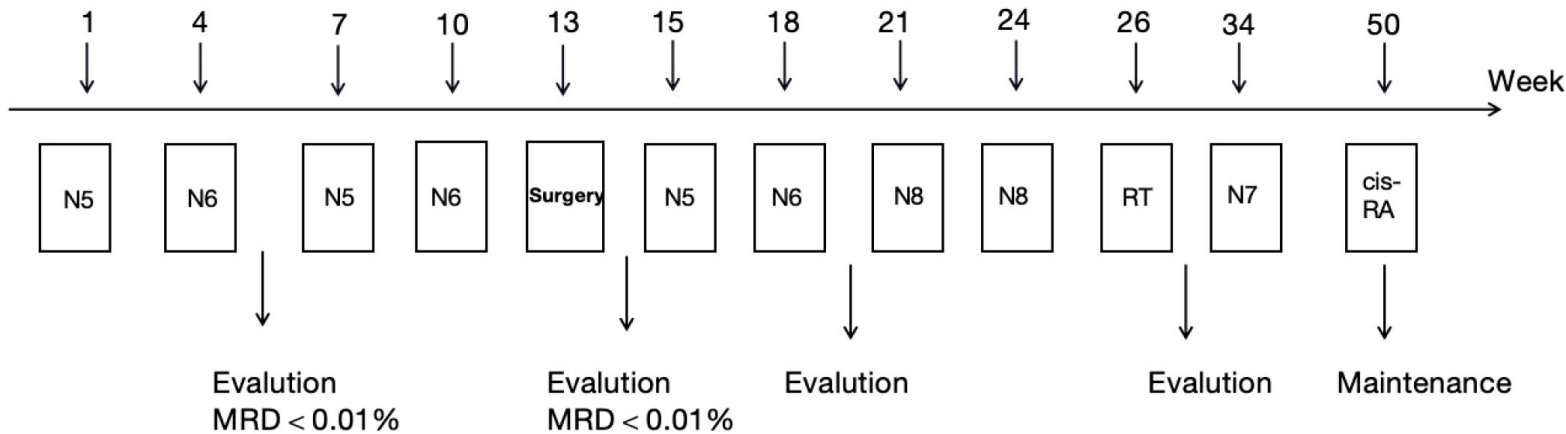
Treatment schedule for neuroblastoma. RT, radiotherapy; 13-cis-RA, 13-cis-retinoic acid.

From October 1, 2019, to December 10, 2019, alternating courses of N5 (specific drug) and N6 (specific drug) chemotherapy were administered for 4 cycles according to the German Pediatric Oncology and Hematology Association (GPOH)-NBL 2017 high-risk protocol[10]. Post-treatment, the left retroperitoneal lesion showed significant reduction (5.0 × 4.2 × 5.8 cm), and the left parietal lesion also decreased markedly (a small nodular shadow approximately 0.7 cm in diameter was observed beneath the inner table of the left parietal bone). On January 4, 2020, the patient underwent complete resection of the left adrenal mass and lymph node dissection. Postoperative pathology revealed chemotherapy-altered left retroperitoneal adrenal neuroblastoma (differentiated type), with 5% tumor cells showing hemorrhage, necrosis, and calcification, and evidence of lymph node metastasis. Immunohistochemistry results were as follows: Syn (+), CgA (+), TH (+), ALK (+); Ki-67 (3%+); N-myc (−), S-100 (−), PHOX2B (+). Whole-genome microarray for pediatric solid tumors showed no MYCN amplification, no loss of heterozygosity (LOH) at the 1p region, no variation at the 11q region, and a diploid chromosome complement. From January 20, 2020, to February 13, 2020, two additional cycles of N5 and N6 chemotherapy were given per the GPOH-NBL-2017 protocol (**Figure 2**; **Table 1**). PET-CT on March 13, 2020, indicated complete metabolic response in para-aortic lymph nodes, multiple right iliac vascular nodules, and systemic skeletal lesions, consistent with tumor response post-treatment. From March 17, 2020, to April 8, 2020, two cycles of N8 chemotherapy were administered. Follow-up contrast-enhanced CT of the head, thorax, and abdomen on April 2020 showed a soft-tissue density nodule at the level of the left renal artery in the para-aortic region, with increased density in previously noted cranial bone destructive areas; no abnormal parenchymal density or enhancement was observed in the brain. After multidisciplinary discussion, abdominal tumor bed radiotherapy (11 fractions of 1.8 Gy each) and cranial metastatic site radiotherapy (12 fractions of 1.8 Gy each) were performed from April 24, 2020, to June 23, 2020(**Table 1**). From June 12, 2020, to March 17, 2021, the patient received one cycle of N7 maintenance chemotherapy and seven cycles of 13-cis-retinoic acid therapy. At the time of diagnosis in early 2019, the patient was treated according to the GPOH-NBL-2017 protocol, which represented the standard frontline therapy for high-risk neuroblastoma at that time. Autologous hematopoietic stem cell transplantation was not performed, as it was declined by the patient’s family for personal reasons. The patient did not receive anti-GD2 monoclonal antibody therapy, as its routine incorporation into first-line consolidation had not yet been established. During this period, minimal residual disease (MRD) monitoring on December 28, 2020, showed <0.01%, and neuron-specific enolase (NSE) levels decreased to a low of 17.24 ng/mL.

**Table 1:** Treatment regimens and doses for neuroblastoma.

Treatments		Single dose	Cumulative dose
Chemotherapy (9 cycles)	Cyclophosphamide	100mg/m^2^/150mg/m^2^	2600mg/m^2^
	Cisplatin	40mg/m^2^	480mg/m^2^
	Etoposide	100mg/m^2^	1400mg/m^2^
	Ifosfamide	1500mg/m^2^	22500mg/m^2^
	Doxorubicin	30mg/m^2^	180mg/m^2^
	Dacarbacine	200mg/m^2^	2400mg/m^2^
	Topotecan	1.0mg/m^2^	21mg/m^2^
	Vincristine	1.5mg/m^2^	9mg/m^2^
	Vindesine	3.0mg/m^2^	9mg/m^2^
Radiotherapy	Abdominal tumor bed	11F/1.8Gy	19.8Gy
	skull metastasis	12F/1.8Gy	21.6Gy

### Development, diagnosis, and treatment of therapy-related AML

**Figure 3:**
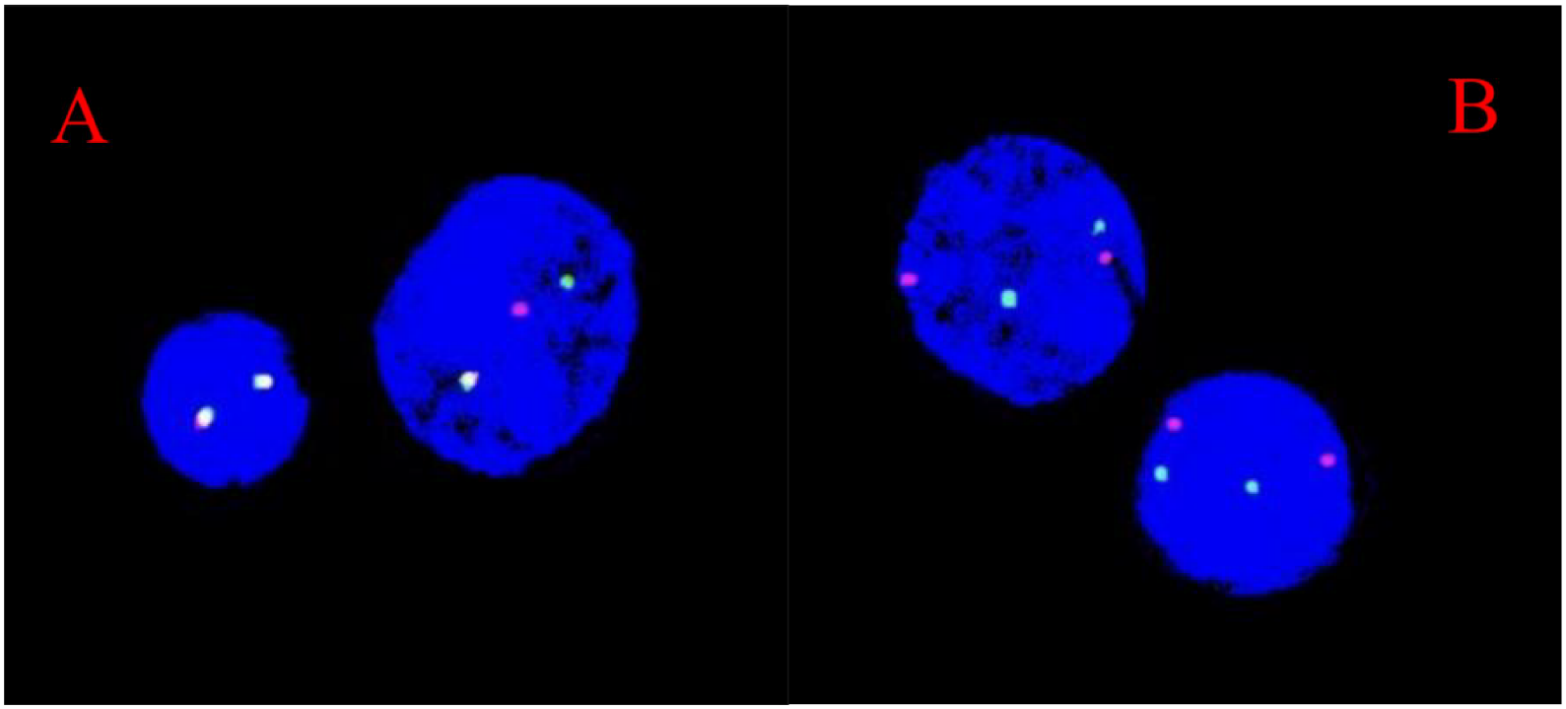
**(A)** MLL probe; target locus: 11q23. **(B)** D7Z1/D7S486 probe; target loci: 7p11.1-q11.1/7q31. The MLL break-apart probe showed separated signals, with a positivity rate of approximately 71%.

On March 19, 2021, the patient developed limb pain without an obvious cause. Bilateral bone marrow smears and neuroblastoma minimal residual disease (NB-MRD) assessments remained negative, serum NSE levels continued to decline, and imaging follow-up showed no evidence of neuroblastoma recurrence. However, peripheral blood examination revealed marked trilineage abnormalities, with a white blood cell count of 165.9 × 10^9/L, circulating blasts accounting for 73%, hemoglobin of 82 g/L, and platelet count of 48 × 10^9/L. Bone marrow examination performed on March 19, 2021, demonstrated acute myelomonocytic leukemia (FAB M4 subtype). Flow cytometric immunophenotyping showed blasts comprising 75.64% of nucleated cells, expressing CD11b, CD13, CD14 (partial), CD15, CD33, CD36, CD38, CD58, CD64, cCD11c, HLA-DR, and myeloperoxidase, supporting the diagnosis of secondary acute myeloid leukemia. Molecular analysis revealed positivity for the MLL/AF9 fusion gene and EVI1 overexpression. FISH analysis demonstrated MLL rearrangement in approximately 71% of interphase cells (**Figure 3**), and conventional cytogenetics showed a karyotype of 46,XX,t (9, 11)(p21;q23). Transcriptome sequencing further identified a KMT2A–MLLT3 fusion and an activating KRAS mutation. The final diagnosis was therapy-related acute myelomonocytic leukemia (M4 subtype, MLL/AF9 and EVI1 positive), classified as high risk. Head MRI performed on March 24, 2021, revealed a nodule in the right frontal region of uncertain nature, with otherwise stable cranial findings (**Figures 1B1–B4**). Cerebrospinal fluid examination on March 29, 2021, was negative for leukemic involvement.

From March 22 to June 25, 2021, the patient received four cycles of intensive chemotherapy according to the CCLG-AML-2019 high-risk protocol (**Table 2**), with one intrathecal chemotherapy administration per cycle. After completion of induction and consolidation therapy, bone marrow morphology, Bone marrow evaluation demonstrated morphological complete remission. However, multiparameter flow cytometry–based MRD assessment (sensitivity 10^-4^) identified residual leukemic cells at 1.21%, whereas MLL/AF9 transcripts were undetectable by quantitative RT-PCR, indicating discordant MRD findings. Following multidisciplinary team discussion, the patient proceeded to allogeneic hematopoietic stem cell transplantation (allo-HSCT) on July 26, 2021. Stem cells were obtained from a healthy unrelated donor identified through the China Marrow Donor Program, with a 10/10 HLA high-resolution match, and peripheral blood stem cells were used as the graft source. The conditioning regimen consisted of decitabine(-12d to -10d, 20mg/m^2^.day), cyclophosphamide(-4d to -3d, 1.8g/m^2^.day), busulfan(-7d to -5d, 1.1mg/kg.per dose, q6h), cytarabine(-9d to -8d, 4g/m^2^.day), and anti-thymocyte globulin (ATG, -4d to -2d, 5mg/kg.day). The infused cell dose was within the standard range for pediatric transplantation (CD34^+^cells >5 × 10^6/kg). Graft-versus-host disease (GVHD) prophylaxis included methotrexate, mycophenolate mofetil, and cyclosporine. Neutrophil engraftment was achieved on day +14, and platelet engraftment on day +23. The patient experienced transient fever around day +7 post-transplant, consistent with engraftment syndrome, which resolved after short-term corticosteroid treatment. No clinically significant acute or chronic GVHD was observed. Full donor chimerism was confirmed after transplantation. A fibroblast-based radio- or chemosensitivity assay was not performed in this patient.

**Table 2:** Treatment regimens and post-treatment evaluation for t-AML.

Time	Regimens	After chemotherapy evaluation			
		BM	MRD	MLL/AF9	EVI1
2021.3.22	DAH (Daunorubicin, 40mg/m^2^.day, d1,3,5 Cytarabine 100mg/m^2^.per dose,q12h,d1–7 Homoharringtonine, 3mg/m^2^.day d1-5)	CR	<0.01%	positive	Highly expressed
2021.4.20	IAH (Idarubicin 40mg/m^2^.day, d1,3,5, Cytarabine 100mg/m^2^.per dose,q12h,d1–7 Homoharringtonine, 3mg/m^2^. day d1-5)	Myeloblasts 3.0% and promonocytes 3.0%	3.23%	positive	Highly expressed
2021.5.28	MA (Doxorubicin 40mg/m2.day, d1,3,5, Cytarabine 2g/m2.per dose,q12h,d1-3 )	Myeloblasts 3.5% and promonocytes 2.5%	2.16%	negative	Highly expressed
2021.6.19	HA Homoharringtonine, 3mg/m2. day d1-7 Cytarabine 1g/m2.per dose,q12h,d1-3)	Myeloblasts 3.0% and promonocytes 2.5%	1.21%	negative	Highly expressed

### Last follow-up

During follow-up, peripheral blood counts normalized, bone marrow examinations consistently demonstrated sustained complete remission, and repeated assessments showed persistently negative bone marrow MRD and NB-MRD. Molecular monitoring revealed undetectable MLL/AF9 transcripts, low EVI1 expression, and no detectable KRAS mutation. At the last follow-up on July 1, 2025, the patient had maintained continuous remission for 51 months after transplantation, was in good general condition, and had returned to regular school activities.

## Discussion

Studies in neuroblastoma (NB) survivors, utilizing data from large cohorts, indicate that the 20-year cumulative incidence of second malignant neoplasms (SMNs) ranges from 5.8% to 9.3% (12). Previous studies have shown that the risk and latency of therapy-related acute myeloid leukemia vary according to the causative therapeutic exposure. In particular, t-AML associated with topoisomerase II inhibitors typically develops within a relatively short latency period of 1–5 years and is frequently characterized by KMT2A (MLL) rearrangements, without a preceding myelodysplastic phase (2). The remarkably short latency of approximately one month between the completion of neuroblastoma therapy and the diagnosis of t-AML in our patient is a striking feature. While the onset of topoisomerase II inhibitor-related t-AML is generally recognized to occur within a shorter window (typically 1–5 years) compared to alkylating agent-associated cases, cases with latencies of less than one year, including several months, are documented in the literature (13, 14). These reports confirm that secondary leukemogenesis can be an early event following cytotoxic exposure, particularly to agents like etoposide. Our case, therefore, resides at the very shortest end of this reported spectrum. This observation underscores a critical clinical implication: the risk for secondary myeloid neoplasms commences immediately after intensive therapy concludes. It challenges the perception of a completely “safe” early post-treatment period and reinforces the necessity for vigilant surveillance and prompt evaluation of concerning symptoms from the very start of follow-up in high-risk neuroblastoma survivors. Secondary malignancies in treated cases of neuroblastoma described in literature includes thyroid tumor, osteogenic sarcoma, soft tissue sarcoma, acute myeloid, lymphoid leukemias and brain tumour (4).

In pediatric cancer survivors, therapy-related myeloid neoplasms are rare but well-recognized late effects, with prior exposure to radiotherapy and topoisomerase II inhibitors being key risk factors; notably, younger age at initial diagnosis has been associated with a shorter latency, which may partly explain the exceptionally early onset observed in our patient (15, 16). Notably, low-risk patients usually only receive surgical therapy; however, they still have a significantly higher probability of developing SMNs than expected, which suggests that genetic factors may be crucially involved in the incidence of SMNs (17).

Therapy-related acute myeloid leukemia (t-AML) is a well-recognized consequence of the mutagenic effects of cytotoxic therapy and encompasses biologically distinct entities depending on the causative agents. Broadly, t-AML can be divided into two major subtypes with differing clinical and genetic characteristics. Alkylating agent–related t-AML typically manifests after a prolonged latency of 5–10 years, is often preceded by myelodysplastic syndrome, and is frequently associated with unbalanced cytogenetic abnormalities such as monosomy 5 or 7. In contrast, t-AML following exposure to DNA topoisomerase II inhibitors develops after a much shorter latency period and is characterized by balanced chromosomal translocations, most commonly involving the KMT2A (MLL) locus at 11q23 (18). The present case is consistent with the latter subtype. The detection of the t (9, 11)(p21;q23) translocation—the most frequent 11q23 rearrangement in pediatric AML—strongly suggests a causal link with prior exposure to etoposide (VP16) during neuroblastoma treatment. KMT2A rearrangements are particularly enriched in therapy-related AML arising after topoisomerase II inhibition and are most often observed in monocytic subtypes (FAB M4/M5). Although certain KMT2A fusion partners, including t (9, 11), have been associated with relatively improved outcomes following allogeneic hematopoietic stem cell transplantation compared with other 11q23 rearrangements, prognosis remains highly heterogeneous. Notably, this patient also demonstrated marked MECOM (EVI1) overexpression, a molecular feature frequently associated with chromosomal damage, treatment-related leukemogenesis, and adverse outcomes. EVI1 encodes a transcription factor essential for normal hematopoiesis, and its aberrant activation in AML has been linked to high relapse rates and reduced overall survival, particularly in the context of 11q23-rearranged disease (19, 20). Given the unusually short latency of t-AML development in this case, the possibility of an underlying genetic cancer predisposition was carefully considered. A detailed family history revealed no malignancies among first- or second-degree relatives. In addition, comprehensive pan-genomic testing performed at the time of the initial neuroblastoma diagnosis did not identify any pathogenic or likely pathogenic germline variants in TP53 or other established hereditary cancer predisposition genes. Genomic profiling of the leukemic blasts demonstrated somatic driver alterations, including a KMT2A–MLLT3 fusion, EVI1 overexpression, and an activating KRAS mutation, which are consistent with an acquired, therapy-related leukemic process. Based on the available clinical and molecular data, no defined germline cancer predisposition syndrome was identified. Nevertheless, we acknowledge that current testing strategies may not fully capture rare, low-penetrance, or as-yet-unrecognized susceptibility variants.

Radiation therapy (RT), as a critical component of neuroblastoma treatment, effectively controls local tumor progression; however, its carcinogenic potential has garnered increasing attention. Studies show that children receiving RT face an elevated risk of secondary solid tumors later in life. This risk becomes significantly pronounced, particularly when the radiation dose exceeds 30 Gy, demonstrating a clear dose-response relationship (21). Current research indicates that children undergoing high-dose chemotherapy (HDCT), especially those receiving radiotherapy exceeding 2340 cGy, exhibit a significantly increased incidence of secondary malignant neoplasms. RT not only exerts a direct cytotoxic effect on tumor cells but also promotes the proliferation and invasiveness of non-irradiated cells by releasing extracellular vesicles that induce survival signals in these bystander cells (22). This release of extracellular vesicles is particularly evident in neuroblastoma cell lines. Following irradiation, these vesicles enhance the survival rate, migratory capacity, and even induce radioresistance in non-irradiated cells, indicating that the effects of RT extend beyond the primary tumor and may potentially initiate new malignancies within the body (23). The overall prevalence of central nervous system (CNS) metastasis in NB is 1.7-11.7%, with generally low occurrence at initial diagnosis (24). The central nervous system (CNS) may act as a sanctuary site for neuroblastoma (NB) because of the limited penetration of many chemotherapeutic agents across the blood–brain barrier (BBB), and effective treatment options for confirmed parenchymal CNS involvement remain limited. Current guidelines recommend irradiation of the primary tumor bed in high-risk NB to reduce the risk of local recurrence; however, the indications for radiotherapy to metastatic sites remain controversial (25). In the present case, the patient initially presented with a calvarial metastatic lesion, with cranial imaging features suggestive of possible dural and/or pial involvement, rather than a definitive parenchymal CNS metastasis. Given the indistinct radiographic boundary between the calvarial lesion and the adjacent cerebral cortex, multidisciplinary discussion considered a potential risk of CNS sanctuary involvement. Consequently, radiotherapy was administered to both the abdominal primary tumor bed and the calvarial lesion. While this approach may have contributed to local disease control, it could also represent a potential contributing factor to the development of the patient’s second malignancy. These findings underscore the importance of individualized radiotherapy decision-making, the application of more precise radiation techniques, and further investigation into optimized combinations of radiotherapy and systemic therapy to balance disease control with long-term toxicity and the risk of secondary neoplasms.

T-MN frequently exhibits resistance to conventional chemotherapy, leading to poorer overall outcomes compared to *de novo* AML, with a median overall survival of only 8–10 months (15, 26, 27). However, our patient has remained disease-free for 50 months following hematopoietic stem cell transplantation and has now returned to school. Therefore, performing hematopoietic stem cell transplantation as soon as possible may be the only means to cure t-AML. Therapy-related AML (t-AML) is a critical treatment complication for various cancers and autoimmune diseases, typically exhibiting high-risk features in over two-thirds of patients. Karyotype analysis and genetic testing can help elucidate the molecular biological features of the disease, thereby aiding in diagnosis and prognostic evaluation. Recent insights into AML’s molecular drivers have transformed risk stratification and enabled biology-targeted, personalized treatment strategies. These developments, including novel targeted therapies, have begun to improve outcomes in t-AML. Current research now prioritizes the discovery of even more effective treatments, with clinical trials actively testing novel agents to boost cure rates (5).

Most t-AML patients exhibit clonal chromosomal abnormalities and fall into the category of refractory leukemia. While pediatric patients typically tolerate relatively higher doses of chemotherapy compared with adults, treatment remains complex because developing organs are particularly susceptible to therapy-related toxicity, necessitating careful balancing of treatment intensity and long-term adverse effects.

The treatment of pediatric t-AML should be tailored to the individual patient’s condition, with careful control of chemotherapy dosage and intensity. Hematopoietic stem cell transplantation represents a potentially curative approach for t-AML.

In recent years, increasing attention has been directed toward balancing therapeutic efficacy with long-term toxicity in high-risk neuroblastoma. Large cohort studies have shown that the incidence of second malignancies is strongly influenced by treatment intensity within risk-adapted therapeutic protocols (28). Accordingly, emerging therapeutic strategies aim to maintain disease control while reducing late adverse effects. One such approach is maintenance therapy with difluoromethylornithine (DFMO) (29), which has shown promise in improving event-free survival with a potentially more favorable long-term toxicity profile. Ongoing refinement of neuroblastoma treatment may therefore have important implications for reducing the burden of therapy-related secondary malignancies in survivors.

## Conclusion

This case illustrates an uncommon but clinically significant secondary malignancy occurring shortly after completion of treatment for high-risk neuroblastoma. The development of therapy-related acute myeloid leukemia with high-risk molecular features highlights the potential vulnerability of neuroblastoma survivors to early secondary hematologic neoplasms following intensive multimodal therapy. Importantly, timely recognition and allogeneic hematopoietic stem cell transplantation resulted in durable long-term remission. These findings underscore the need for early surveillance for secondary malignancies and individualized treatment strategies in pediatric neuroblastoma survivors to optimize long-term outcomes.

## Data Availability

The raw data supporting the conclusions of this article will be made available by the authors, without undue reservation.
